# The spectrum of renal thrombotic microangiopathy in lupus nephritis

**DOI:** 10.1186/ar4142

**Published:** 2013-01-15

**Authors:** Di Song, Li-hua Wu, Feng-mei Wang, Xiao-wei Yang, Di Zhu, Min Chen, Feng Yu, Gang Liu, Ming-hui Zhao

**Affiliations:** 1 Renal Division, Department of Medicine, Peking University, First Hospital, Peking University Institute of Nephrology, Key Laboratory of Renal Disease, Ministry of Health of China, Key Laboratory of Chronic Kidney Disease Prevention and Treatment, Ministry of Education of China, Beijing 100034, PR China; 2 Department of Nephrology, General Hospital of Ningxia Medical University, Ningxia 750004, PR China

## Abstract

**Introduction:**

Among various lupus renal vascular changes, thrombotic microangiopathy (TMA) presented with the most severe clinical manifestations and high mortality. The pathogenesis of TMA in systemic lupus erythematosus (SLE) was complicated. The aim of this study was to assess clinical manifestations, laboratory characteristics, pathological features and risk factors for clinical outcomes of lupus nephritis patients co-existing with renal TMA in a large cohort in China.

**Methods:**

Clinical and renal histopathological data of 148 patients with biopsy-proven lupus nephritis were retrospectively analyzed. Serum complement factor H, A Disintegrin and Metalloprotease with Thrombospondin type I repeats 13 (ADAMTS-13) activity, antiphospholipid antibodies and C4d deposition on renal vessels were further detected and analyzed.

**Results:**

In the 148 patients with lupus nephritis, 36 patients were diagnosed as co-existing with renal TMA based on pathological diagnosis. Among the 36 TMA patients, their clinical diagnoses of renal TMA were as followings: 2 patients combining with thrombotic thrombocytopenic purpura-hemolytic uremic syndrome, 2 patients combining with anti-phospholipid syndrome, 2 patients with malignant hypertension, 1 patient with scleroderma and the other 29 patients presenting with isolated renal TMA. Compared with the non-renal TMA group, patients with renal TMA had significantly higher urine protein (7.09 ± 4.64 vs. 4.75 ± 3.13 g/24h, *P *= 0.007) and serum creatinine (159, 86 to 215 vs. 81, 68 to 112 μmol/l, *P *<0.001), higher scores of total activity indices (AI) (*P *<0.001), endocapillary hypercellularity (*P *<0.001), subendothelial hyaline deposits (*P *= 0.003), interstitial inflammation (*P *= 0.005), glomerular leukocyte infiltration (*P *= 0.006), total chronicity indices (CI) (*P *= 0.033), tubular atrophy (*P *= 0.004) and interstitial fibrosis (*P *= 0.018). Patients with renal TMA presented with poorer renal outcome (*P *= 0.005) compared with the non-TMA group. Renal TMA (hazard ratio (HR): 2.772, 95% confidence interval: 1.009 to 7.617, *P *= 0.048) was an independent risk factor for renal outcome in patients with lupus nephritis. The renal outcome was poorer for those with both C4d deposition and decreased serum complement factor H in the TMA group (*P *= 0.007).

**Conclusions:**

There were various causes of renal TMA in lupus nephritis. Complement over-activation via both classical and alternative pathways might play an important role in the pathogenesis of renal TMA in lupus nephritis.

## Introduction

Renal involvement is common in systemic lupus erythematosus (SLE) [1]. In addition to glomerulonephritis, the status of renal vascular lesions is also important in lupus nephritis because their presence can adversely affect the prognosis of the renal disease [2,3]. Among various lupus renal vascular changes, thrombotic microangiopathy (TMA) presented with the most severe clinical manifestations and high mortality [4]. Since the pathogenesis of TMA in lupus nephritis is complex and unclear, detailed descriptions about it were lacking in the literature.

In fact, TMA in lupus nephritis consisted of a group of diseases, including anti-phospholipid syndrome (APS), thrombotic thrombocytopenic purpura-hemolytic uremic syndrome (TTP-HUS), scleroderma, malignant hypertension and calcineurin inhibitor-associated thrombotic microangiopathy and so on. The pathogenesis of TMA in SLE was complicated. Recently, Danielle *et al. *demonstrated that activation of the complement classical pathway might be a crucial factor in the development of TMA in lupus nephritis [5].

The aim of this study was to assess clinical manifestations, laboratory characteristics, pathological features and risk factors for clinical outcomes of patients with TMA in lupus nephritis in a large cohort of Chinese patients. The roles of A Disintegrin and Metalloprotease with Thrombospondin type I repeats 13 (ADAMTS-13), complement factor H, antiphospholipid antibodies and C4d deposition in renal vessels were further evaluated.

## Methods

### Patients

Clinical and renal histopathological data of 148 patients with renal biopsy-proven lupus nephritis, diagnosed between May 2002 and July 2008 in Peking University First Hospital were reviewed.

### Clinical evaluation and definitions of the diseases and lesions

All the included patients fulfilled the 1997 American College of Rheumatology revised criteria for SLE [6]. The disease activity was assessed by the Systemic Lupus Erythematosus Disease Activity Index (SLEDAI) [7,8].

TTP-HUS was characterized by microangiopathic hemolytic anemia and thrombocytopenia and/or fever and/or acute renal impairment and/or neurologic impairment. APS was defined by the Sapporo criteria [9].

Renal TMA was defined as interlobular artery, arteriole and glomerular capillary lesions, including endothelial cell swelling, lumen narrowing or obliteration and thrombi formation by light microscopy. Swelling of glomerular endothelial cells, detachment from the glomerular basement membrane and widening of the subendothelial space were identified by electron microscopy (Additional file 1, Figure S1A, B). No striated fine fibrillary structure or other specific structure of cryoglobulinemic glomerulonephritis was identified by electron microscopy. The lesions were divided into acute changes and chronic changes [5]. The acute lesion was defined as the presence of at least one fibrin microthrombus (conformed by fibrin and CD61 staining), either in glomeruli, or in small arterioles and/or arteries (Additional file 2, Figure S2A, B). Chronic changes were mucoid changes and onion skin lesions of arterioles and/or arteries (Additional file 2, Figure S2C).

The response to therapy includes complete remission, partial remission and treatment failure detailed in previous works [10-13]. The indications for plasma exchange was as follows: lupus nephritis with crescentic glomerulonephritis, fibrinoid necrosis and thrombotic microangiopathy in renal pathological changes, severe extra-renal involvement, such as central nervous system injury, hematological abnormalities, cardiovascular diseases, catastrophic antiphospholipid antibody syndrome and so on [14].

A relapse was defined as: 1) nephritic relapse: a recent increase of serum creatinine by >50% with active urinary sediments; 2) proteinuric relapse: development of either a nephrotic syndrome (proteinuria >3.5 g/day and serum albumin <30 g/L) or proteinuria >1.5 g/day without other causes, in previously non-proteinuric patients [15,16].

The patients were followed up in outpatient clinics specified for lupus nephritis. The primary end point was defined as death and the secondary end point was defined as end stage renal disease (ESRD) or doubling of serum creatinine.

### Laboratory assessment

The following laboratory features were further detected using serum at the day of renal biopsy.

Serum antinuclear antibodies (ANA) were detected using indirect immunofluorescence assay (EUROIMMUN, Lübeck, Germany) and anti-double-stranded DNA (ds-DNA) antibodies were detected using Crithidia luciliae indirect immunofluorescence test (EUROIMMUN, Lübeck, Germany). Anti-extractable nuclear antigen (ENA) antibodies, including anti-Sm, anti-SSA, anti-SSB and anti-RNP antibodies, were detected using immunodotting assay (EUROIMMUN, Lübeck, Germany). Anti-cardiolipin antibodies and anti-β2GP-1 antibodies were detected using enzyme-linked immunosorbent assay (ELISA) (EUROIMMUN, Lübeck, Germany). Serum C3 was determined using rate nephelometry assay (Beckman-Coulter, IMMAGE, Brea, California, USA, normal range >0.85 g/L). Serum cryoglobulins were detected by spectrophotometry (Beckman-Coulter, IMMAGE, Brea, California, USA).

#### Quantification of serum complement factor H

The method to detect serum complement factor H (CFH) was the same as previously described [17], with mild modification. Serial concentrations of commercial available highly purified human factor H from 1,050 μg/ml to 16.4 μg/ml were used to develop the standard curve. The CFH level of each sample was calculated using Curve expert 1.3 (Hyams DG, Starkville, Mississippi, USA). The linear portion of the curve was subsequently used for the measurement of serum factor H. All assays were run in duplicate, and when standard errors were over 10%, samples were routinely re-analyzed.

#### Detection of serum ADAMTS-13 activity

The ADAMTS-13 activity assay was the same as previously described [18]. Data were analyzed as the percentage of collagen-binding activity remaining after dialysis compared to the collagen binding activity in the individual's baseline sample. One hundred percent minus the residual collagen-binding activity was arbitrarily regarded as the ADAMTS-13 activity. Inter-assay precision was determined by evaluating single normal human plasma in 10 consecutive assay runs for ADAMTS-13 activity determination. The inter-assay percent coefficient of variation was found to be <10%.

### Routine renal histopathology

The renal biopsy specimens were routinely examined by light microscopy, direct immunofluorescence and electron microscopy techniques.

Lupus nephritis was re-classified according to the International Society of Nephrology and Renal Pathology Society (ISN/RPS) 2003 lupus nephritis classification system [19].

#### Light microscopy examination

Renal biopsy specimens were fixed in 4.5% buffered formaldehyde for light microscopy. Consecutive serial 3 μm sections were used for histological staining. Stains employed included hematoxylin and eosin (H&E), periodic acid-Schiff (PAS), silver methenamine (Meth) and Masson's trichrome. Pathological parameters, such as activity indices (AI) and chronicity indices (CI), were approached by renal pathologists using a modification of a previously reported system involving semi-quantitative scoring of specific biopsy features [20,21].

#### Direct immunofluorescence examination

The direct immunofluorescence for immunoglobulin G (IgG), immunoglobulin A (IgA), immunoglobulin M (IgM), C3, C1q and fibrin was semi-quantitatively graded from 0 to 4 according to the intensity of fluorescence, respectively.

#### Electron microscopy examination

Renal biopsy specimens were fixed in 2.5% paraformaldehyde for electron microscopy. After being embedded in epon, ultrathin sections were mounted on metal grids and stained with uranyl acetate before being viewed in a transmission electron microscope (JEM-1230; JEOL, Tokyo, Japan).

### C4d staining on renal vessels by immunohistochemistry

Staining of C4d on renal vessels was performed by immunohistochemistry as was previously described [22]. Rabbit anti-human C4d polyclonal antibodies (Abcam, Cambridge, UK) were used as primary antibodies (dilution 1:400). As negative controls, primary antibodies were replaced by normal rabbit IgG. The sections were examined by light microscopy (Additional file 2, Figure S2D).

### CD61 staining in kidneys by immunohistochemistry

The method of CD61 staining was similar to C4d [23]. Rabbit anti-human CD61 polyclonal antibodies (Zhongshan Golden Bridge Biotechnology, Beijing, China) were used as primary antibodies (dilution 1:200).

### Blood samples

Sera were obtained from peripheral blood at the same day as the renal biopsy before initiation of immunosuppressive treatment. All sera samples were stored at -80°C until used. Repeated freeze/thaw cycles were avoided.

Informed consent was obtained for blood sampling and renal biopsy from each patient. The research was in compliance with the Declaration of Helsinki. The design of this work was approved by the local ethical committees of Peking University First Hospital (No. 2012[470]).

### Statistical analysis

Statistical software SPSS 18.0 (SPSS, Chicago, IL, USA) was used for statistical analysis. Quantitative data were expressed as mean ± SD, and median with range (minimum, maximum). For comparison of clinical and pathological features of patients, the Student's *t*-test, one-way ANOVA analysis of variance and Chi-square test were used. Kaplan-Meier curves were used to analyze the patients' prognoses. Survival analysis was performed using the log-rank test. Multivariate analysis with Cox regression was used to determine the prognostic factors. Results were expressed as hazard ratio (HR) with 95% confidence intervals. Statistical significance was considered as *P *<0.05.

## Results

### General data of patients with renal TMA in lupus nephritis

First, we analyzed the general data of patients with renal TMA in lupus nephritis (details in Table 1). Among the 148 lupus nephritis patients enrolled in this study, 36 were identified as combining with renal TMA changes by pathological findings. In the TMA group, the average age was 29.75 ± 9.24 (15 to 52) at presentation. Ten (27.8%) patients were male and 26 (72.2%) were female, with a male to female ratio of 1:2.6. Compared with previous studies, the ratio of renal TMA in lupus nephritis was higher.

**Table 1:** General data of patients combined with renal TMA and lupus nephritis

No.	Age (years)	Clinical diagnosis	Pathological types	CFH (μg/ml)	ACL	Anti-β2GP-1	ADAMTS-13 Activity (%)	C4d deposition on vascular wall
1	25	LN	IV-G(A/C)	386.0	**-**	**-**	88	**-**
2	38	LN	IV-G(A/C)	670.2	**-**	**-**	83	**++**
3	34	LN	IV-G(A)	166.8	**-**	**-**	70	**+**
4	45	LN+MHT	IV-G(A/C)	694.8	**-**	**-**	68	**-**
5	28	LN	IV-G(A/C)	298.8	**-**	**-**	94	**+**
6	43	LN	IV-G(A)+V	642.8	**-**	**-**	75	**-**
7	26	LN+Scleroderma	IV-G(A)	136.0	**-**	**-**	74	**+**
8	26	LN	IV-G(A)+V	112.8	**-**	**-**	95	**-**
9	23	LN	IV-G(A)+V	385.4	**-**	**-**	91	**+**
10	27	LN+TTP-HUS	IV-G(A/C)	146.0	**-**	**-**	97	**+**
11	32	LN	III(A/C)+V	467.6	**-**	**-**	95	**+**
12	38	LN+MHT	IV-G(A/C)	251.6	**-**	**-**	98	**-**
13	20	LN	IV-G(A)	362.4	**-**	**-**	98	**-**
14	31	LN	IV-G(A)	551.2	**-**	**-**	71	**-**
15	25	LN	IV-G(A)	567.4	**-**	**-**	85	**+**
16	19	LN	IV-G(A)	620.4	**-**	**-**	98	**+**
17	34	LN+TTP-HUS	IV-G(A)	365.6	**-**	**-**	89	**-**
18	19	LN	IV-S(A)	457.0	**-**	**-**	89	**-**
19	31	LN	IV-G(A)	491.4	**-**	**-**	97	**-**
20	18	LN+APS	IV-G(A)+V	162.0	**+**	**-**	45	**+**
21	30	LN+APS	V	707.2	**-**	**+**	98	**+**
22	43	LN	III(A/C)+V	498.8	**-**	**-**	96	**+**
23	24	LN	IV-G(A/C)	222.4	**-**	**-**	96	**+**
24	35	LN	IV-G(A/C)	540.0	**-**	**-**	99	**-**
25	40	LN	IV-G(A/C)	117.8	**-**	**-**	98	**-**
26	21	LN	III	201.2	**-**	**-**	63	**+**
27	38	LN	IV-G(A)	672.6	**-**	**-**	88	**+**
28	52	LN	IV-G(A/C)	131.0	**-**	**-**	96	**-**
29	37	LN	V	340.0	**-**	**-**	32	**-**
30	27	LN	IV-G(A)	124.4	**-**	**-**	95	**-**
31	18	LN	IV-G(A/C)	492.8	**-**	**-**	95	**-**
32	15	LN	IV-G(A)	221.2	**-**	**-**	97	**++**
33	33	LN	IV-G(A)	374.0	**-**	**-**	80	**+**
34	15	LN	IV-G(A)	407.2	**-**	**-**	68	**+**
35	40	LN	V	434.2	**-**	**-**	96	**-**
36	20	LN	IV-G(A)	627.6	**-**	**-**	97	**+**

Note: The normal range of serum CFH was 561.3 ± 179.7 (381.6 to 741.0) μg/ml. The normal range of ADAMTS-13 activity was 95% (42% to 99%). Abbreviations: APS, anti-phospholipid syndrome; LN, lupus nephritis; MHT, malignant hypertension; TTP-HUS, thrombotic thrombocytopenic purpura-hemolytic uremic syndrome

Then, the detailed renal pathological data were analyzed. According to the 2003 classification of lupus nephritis, 3 patients were classified Class III (8.3%, including 2 as Class III + V), 30 as Class IV (83.3%, 1 as Class IV-segmental (IV-S) (2.8%) and 25 as Class IV-global (IV-G) (69.4%), including 4 as Class IV-G + V) and 3 as Class V (8.3%). There was no case of Class I, Class II or Class VI in this study. Class III and IV were further subdivided into an active (A) group, active/chronic (A/C) group and chronic (C) group. Within Class III, the number of III (A) was 1, III (A/C) was 2 and III (C) was 0. The Class IV-S is IV-S (A). Within Class IV-G, the number of IV-G (A) was 18, IV-G (A/C) was 12 and IV-G (C) was 0. For further evaluation of renal pathological features of TMA, 17 patients presented with pure acute lesions, 6 with pure chronic lesions and 13 with both acute and chronic lesions, as described in the Methods. Among the 30 patients with acute lesions, 14 had micro-thrombi in glomeruli and 23 had micro-thrombi in small arterioles and/or arteries. The active and chronic lesions both existed in these patients.

We further analyzed the possible causes of renal TMA in lupus nephritis. Among the 36 patients, there were several clear clinical reasons for renal TMA as follows: 2 patients combining with TTP-HUS, 2 patients combining with APS, 2 patients with malignant hypertension, 1 patient with scleroderma and the other 29 patients presenting with isolated renal TMA. The other potential causes of renal TMA included 19 patients with C4d deposition on renal vascular walls, 2 patients with decreased ADAMTS-13 activity, and 17 patients with decreased serum complement factor H. The ratio of patients with C4d deposition or decreased serum factor H was high. There was no significant correlation between the two risk factors (r = 0.088, *P *= 0.610). All of the 36 patients with renal TMA were serum cryoglobulin negative.

The treatment algorithm was listed as follows. All of the patients received oral prednisone therapy (0.8 to 1 mg/kg/d or equivalent for four to six weeks and tapered slowly to a maintenance dose of 5 to 10 mg/d). A total of 19 patients received plasma exchange, including 2 patients with TTP-HUS, 1 with catastrophic antiphospholipid antibody syndrome, 3 with lupus encephalopathy, 13 with severe crescentic glomerulonephritis, fibrinoid necrosis and thrombotic microangiopathy in renal pathological changes, and 26 patients received methylprednisolone pulse therapy. The majority of patients completed treatment with oral cyclophosphamide (5/36) or monthly intravenous cyclophosphamide (600 to 800 mg/month) (25/36). The other patients received mycophenolate mofetil (3/36), leflunomide (2/36) and azathioprine (1/36). Twenty patients achieved clinical remission, 8 with complete remission and 12 with partial remission. Sixteen patients presented with treatment failure. Most of the patients received immunosuppressive therapy, but the responses to the treatment were not satisfactory.

### Clinical and laboratory parameters

We further compared the clinical and laboratory characteristics of patients with and without renal TMA in lupus nephritis. The clinical and laboratory features of patients in the two groups were listed in Tables 2 and 3. The patients in the renal TMA group were younger than the patients in the non-renal TMA group (29.75 ± 9.24 vs. 34.38 ± 11.95 years, *P *= 0.035) at the time of biopsy. There was a significantly higher ratio of nephrotic syndrome in the renal TMA group than in the non-renal TMA group (83.3% vs. 58.9%, *P *= 0.008). In laboratory findings, there were significantly higher urine protein levels (7.09 ± 4.64 vs. 4.75 ± 3.13 g/24 h, *P *= 0.007) and higher serum creatinine levels (159, 86 to 215 vs. 81, 68 to 112 μmol/l, *P *<0.001) in the renal TMA group compared with patients in the non-renal TMA group. The TMA group presented with more severe renal injury than the control group.

**Table 2:** Comparison of clinical data between lupus nephritis patients with and without renal TMA

	LN with renal TMA	LN without renal TMA	*P*-value
Number of patients	36	112	
Age (mean ± SD)(years)	29.75 ± 9.24	34.38 ± 11.95	0.035
Gender (male/female)	10/26	16/96	0.064
Number of fever (non-infection) (%)	10 (27.8)	33 (29.5)	0.846
Number of neurologic disorder (%)	3 (8.3)	8 (7.1)	1.0
Number of anemia (%)	28 (77.8)	79 (70.5)	0.398
Number of thrombocytopenia (%)	15 (41.7)	30 (26.8)	0.091
Number of hematuria (%)	31 (86.1)	87 (77.7)	0.274
Number of nephrotic syndrome (%)	30 (83.3)	66 (58.9)	0.008
SLEDAI (mean ± SD)	17.02 ± 5.60	17.62 ± 5.68	0.668

**Table 3:** Comparison of laboratory data between lupus nephritis patients with and without renal TMA

	LN with renal TMA	LN without renal TMA	*P*-value
Number of patients	36	112	
Hemoglobin (mean ± SD) (g/l)	92.33 ± 22.03	101.29 ± 25.29	0.059
Urine protein (mean ± SD) (g/24hours)	7.09 ± 4.64	4.75 ± 3.13	0.007
Serum creatinine (median; inter-quartile range) (μmol/l)	159.00 86.00, 215.00	81.50 68.00, 111.50	<0.001
Number of positive ANA (%)	36 (100)	111 (99.1)	1.0
Number of positive anti-dsDNA (%)	28 (77.8)	85 (75.9)	0.817
Number of positive anti-cardiolipin (%)	1 (2.8)	7 (6.2)	0.680
Number of positive anti-β2 GP-I (%)	1 (2.8)	8 (7.1)	0.688
C3 (mean ± SD) (mg/ml)	0.51 ± 0.29	0.59 ± 0.34	0.210

### Renal histopathological evaluation

Renal pathological parameters were then compared between the two groups. The distribution of renal pathological types and the characteristics of renal histopathology of the two groups were listed in Table 4. The proportion of Class IV was significantly higher in the renal TMA group than that in the non-renal TMA group (83.3% vs. 58.0%, *P *= 0.006). In comparison with the non-renal TMA group, patients with renal TMA had significantly higher scores of total AI (*P *<0.001), endocapillary hypercellualrity (*P *<0.001), subendothelial hyaline deposits (*P *= 0.003), interstitial inflammation (*P *= 0.005), glomerular leukocyte infiltration (*P *= 0.006), total CI (*P *= 0.033), tubular atrophy (*P *= 0.004) and interstitial fibrosis (*P *= 0.018), respectively. Also, the results indicated more active and chronic renal involvement in patients with renal TMA.

**Table 4:** Comparison of renal pathological data between lupus nephritis patients with and without renal TMA

	LN with renal TMA	LN without renal TMA	*P*-value
Number of biopsies	36	112	
Class II (%)	0 (0)	5 (4.5)	0.336
Class III (%)	3 (8.3)	26 (23.2)	0.05
Class IV (%)	30 (83.3)	65 (58.0)	0.006
Class V (%)	3 (8.3)	16 (14.3)	0.567
AI score (mean ± SD)	10.78 ± 4.16	7.58 ± 4.29	<0.001
Endocapillary hypercellualrity (mean ± SD)	2.78 ± 0.59	2.20 ± 0.99	<0.001
Cellular crescents (mean ± SD)	2.06 ± 1.94	1.38 ± 1.8	0.055
Karyorrhexis/fibrinoid necrosis (mean ± SD)	1.28 ± 1.06	0.95 ± 1.18	0.137
Subendothelial hyaline deposits (mean ± SD)	1.72 ± 1.09	1.10 ± 1.07	0.003
Interstitial inflammation (mean ± SD)	1.63 ± 0.83	1.19 ± 0.72	0.005
Glomerular leukocyte infiltration (median; inter-quartile range)	1 1, 2	1 0, 1	0.006
CI score (mean ± SD)	3.67 ± 1.91	2.80 ± 2.15	0.033
Glomerular sclerosis (mean ± SD)	0.53 ± 0.70	0.48 ± 0.70	0.733
Fibrous crescents (median; inter-quartile range)	0 0, 1	0 0, 0	0.166
Tubular atrophy (mean ± SD)	1.47 ± 0.61	1.06 ± 0.77	0.004
Interstitial fibrosis (mean ± SD)	1.36 ± 0.59	1.02 ± 0.79	0.018

### Treatment and outcome

Lastly, the therapy algorithm and long-term outcomes were analyzed between the two groups. The treatment and outcomes of renal TMA and non-renal TMA patients were detailed in Table 5. The ratios of patients using plasma exchange and methylprednisolone pulse were significantly higher in TMA group than that in non-TMA group (*P *<0.001, *P *<0.001, respectively). The renal TMA patients presented with significant lower ratios of partial remission (33.3% vs. 58.0%, *P *= 0.01) and higher ratios of treatment failure (44.4% vs. 15.2%, *P *<0.001) compared with the non-renal TMA group.

**Table 5:** Comparison of treatment data between lupus nephritis patients with and without renal TMA

	LN with renal TMA	LN without renal TMA	*P-*value
Number of patients (%)	36	112	
Treatment			
PE (Number of patients (%))	19 (52.8)	7 (6.25)	<0.001
MP (Number of patients (%))	26 (72.2)	18 (16.1)	<0.001
P	36 (100)	112 (100)	1
CYC	30 (83.3)	88 (78.6)	0.536
AZA	1 (2.8)	5 (4.5)	1
MMF	3 (83.3)	11 (9.8)	1
LEF	2 (5.6)	8 (7.1)	1
Treatment response			
CR	8 (22.2)	30 (26.8)	0.586
PR	12 (33.3)	65 (58.0)	0.01
TF	16 (44.4)	17 (15.2)	<0.001
Duration of follow-up (m)	53 (6,240)	53 (6,282)	0.15
Relapse rate	4 (4/20, 20%, 3 with nephritic relapse and 1 with proteinuric relapse)	17 (17/95, 17.89%, 15 with nephritic relapse and 2 with proteinuric relapse)	0.543

AZA, azathioprine; CR, complete remission; CYC, cyclophosphamide; LEF, leflunomide; MMF, mycophenolate mofetil; MP, methylprednisolone impulse; P, oral prednisone; PE, plasma exchange; PR, partial remission; TF, treatment failure

In our cohort study, the lupus patients with renal TMA were followed up for a period of 53 ± 64 months (range 6 to 240 months). The patients without renal TMA were followed up for a period of 53 ± 44 months (range 6 to 282 months). During the similar follow-up time, the relapse rate showed no significant difference between the two groups.

**Figure 1:**
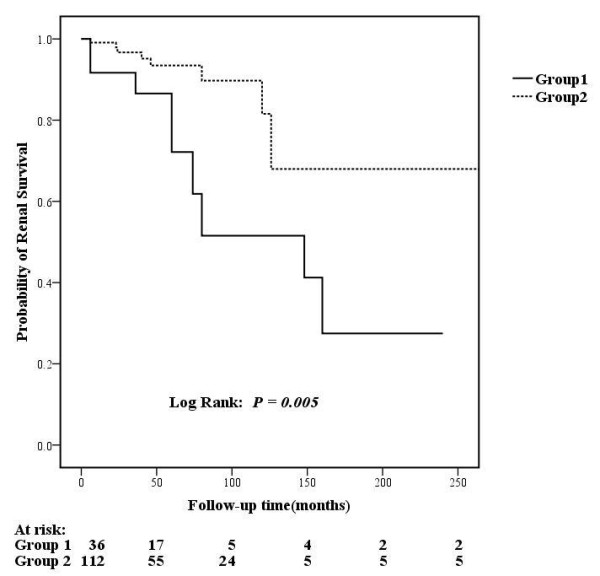
**Comparison of renal outcomes between lupus nephritis patients with and without renal TMA**.

The long-term survival was similar between the two groups. But the renal outcome was significantly poorer in the TMA group (*P *= 0.005, Figure 1). In the TMA group (Group 1), no patients died or reached ESRD, but 10 patients reached the doubling of serum creatinine. In the non-TMA group (Group 2), one patient died, no patient reached ESRD and seven patients reached the doubling of serum creatinine.

**Figure 2:**
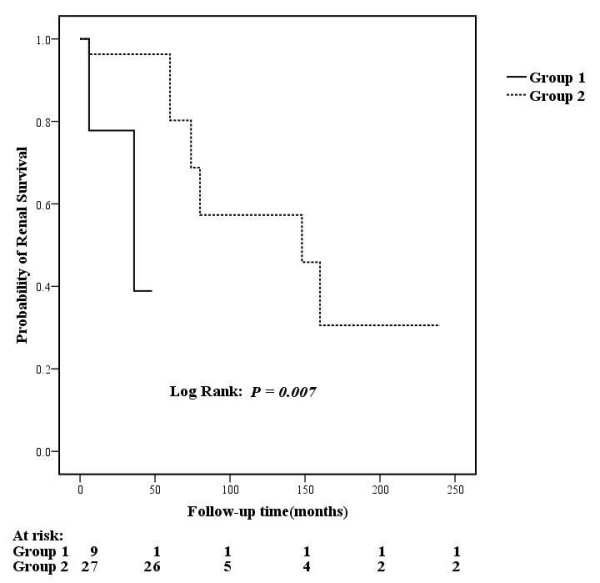
**Comparison of renal outcomes between subgroups of patients with renal TMA**.

Using the log-rank test for univariate survival analysis of renal prognosis in all the patients with lupus nephritis, we found that renal TMA was a risk factor for renal outcome in lupus nephritis (*P *= 0.009). Other univariate risk factors included sex (male, *P *= 0.01), serum creatinine value (*P *<0.001), proteinuria (*P *= 0.032), total activity indices score (*P *= 0.001) and total chronicity indices score (*P *<0.001) (Details in Table 6). Multivariate analysis revealed that renal TMA (HR: 2.772, 95% confidence interval: 1.009 to 7.617, *P *= 0.048) and serum creatinine value (HR: 1.003, 95% confidence interval: 1.002 to 1.005, *P *<0.001) were independent prognostic factors for renal survival (Additional file 3, Table S1). With further analysis, we found that the patients with both C4d deposition and decreased serum factor H (Group 1) presented with higher pathological AI scores (13.44 ± 3.78 vs. 9.89 ± 3.95, *P *= 0.024) and poorer renal outcome (*P *= 0.007, Figure 2) compared with those without the combination in the TMA group (Group 2).

**Table 6:** Univariate survival analysis of patients' renal prognosis with lupus nephritis

	HR	95% confidence interval		*P*-value
Age	0.947	0.896	1.002	0.057
Sex	0.252	0.089	0.717	0.010
Proteinuria	1.109	1.009	1.220	0.032
Serum creatinine value	1.003	1.002	1.005	<0.001
ANA	0.049	0.000	0.000	0.912
Anti-ds-DNA antibody	0.970	0.353	2.664	0.953
SLEDAI	0.961	0.885	1.043	0.344
Activity indices (AI) score	1.220	1.080	1.378	0.001
Chronicity indices (CIs) score	1.428	1.191	1.713	<0.001
Renal TMA	0.275	0.105	0.721	0.009
Anti-cardiolipin antibody	23.596	0.012	45,236	0.412
anti-β2GP-1 antibody	25.022	0.001	647,548	0.535
ADAMTS13 activity	1.542	0.161	14.764	0.707
Complement factor H	0.998	0.996	1.001	0.247
Vascular C4d deposition	0.815	0.320	2.078	0.669

Taking the above results together, although with more intensive immunosuppressive therapy, patients with TMA had a poorer renal outcome, especially those with both C4d deposition and decreased serum factor H, than those without renal TMA.

## Discussion

The prevalence of renal TMA, including acute and chronic changes, in our cohort with lupus nephritis was 24.3% (36/148), which was higher than that in previous studies (0.5% to 10%) [2,24]. The most possible reason might be that our diagnosis was based on the strict histopathological criteria, not just clinical evaluation, which might increase the ratio. In fact, diagnosis of TMA in SLE is sometimes difficult because these two disorders share similar clinical features, including anemia, thrombocytopenia, neurological deficits, renal involvement and fever. Therefore, the pathological criteria should be regarded as the "gold standard" in patients with SLE.

As reported in the previous studies [25-27], patients with both renal TMA and lupus nephritis in our center presented with more severe renal injury features, including a higher amount of proteinuria, higher value of serum creatinine, higher scores of total AI indices, endocapillary hypercellualrity, cellular crescents, subendothelial hyaline deposits, interstitial inflammation, glomerular leukocyte infiltration, total CI indices, tubular atrophy and interstitial fibrosis in pathological evaluations, compared with the patients without TMA. Although with more intensive immunosuppressive therapy, patients with TMA had a poorer renal outcome than those without renal TMA. Renal TMA was found as an independent risk factor for renal outcome in lupus nephritis.

The pathogenesis of renal TMA in lupus nephritis remains unclear and may be multifactorial, which might be attributed to APS, TTP-HUS, malignant hypertension, pregnancy, scleroderma, drugs and so on. Thus, we further investigated the above risk factors in the 36 renal TMA patients. Interestingly, only seven patients were found with clear reasons, including two with TTP-HUS, two with APS, two with malignant hypertension and one with scleroderma. The other 29 patients only presented with pathological evidence of renal TMA.

It is suggested that immune complex-mediated complement activation via the classical pathway plays a key role in the pathogenesis of tissue injury in lupus nephritis [28-30]. C4d is produced mainly through the classical complement activation cascade and can covalently bind to glomerular endothelial surfaces and basement membranes through the thiol ester site [31]. Recently, Danielle *et al. *[5] and Shen *et al. *[32] found that positive C4d staining in glomeruli correlated with the development of renal microthrombi and demonstrated that activation of the complement classical pathway might be a crucial factor in the development of TMA in lupus nephritis. In our study, we also found that a high ratio (19/36) of patients had C4d deposition on vessels in patients with renal TMA. Li *et al. *[33] recently reported that C4d deposition in peritubular capillaries was closely related with low serum C4 level and higher disease activity of lupus nephritis. It is possible that although it intend to clear the immune complexes, the activation of complement classical pathway may further cause the inflammation and injury of the endothelium. Because C4d is also involved in the lectin pathway, we cannot exclude the possibility that C4d deposition partly reflects activation of the mannose-binding lectin (MBL) pathway, which needs further investigation.

Although it has been suggested that the development of SLE, especially lupus nephritis, is closely associated with immune complex-induced complement activation via classical pathways, recent studies [34] demonstrated that activation of the alternative complement pathway could accurately reflect disease activity and the ongoing activation paralleled with flares in patients with SLE, and the alternative pathway might play an important role in complement activation-induced self-injury and inflammatory response in SLE [35]. Further studies suggested that patients with deficiency of complement regulators, such as complement factor H, of the alternative pathway, was susceptible to SLE [36-38]. A recent study has shown that factor H deficiency accelerates the development of lupus nephritis in lupus-prone mice MRL-lpr [39]. As mutations and single nucleotide polymorphisms (SNPs) in complement factor H have been implicated in a variety of human pathological conditions, especially atypical hemolytic uremic syndrome (aHUS), one of the reasons for TMA, we further detected concentrations of serum factor H in our patients. Interestingly, nearly half of the patients in our renal TMA group were found with decreased serum complement factor H. Complement factor H is a fluid phase complement regulator of the alternative complement pathway [40,41]. It can bind to endothelial cells and protects them from being attacked by the complement system. It is suggested that dysfunction or reduced levels of serum factor H may cause endothelial cell damage which may be followed by platelet consumption, red cell damage and the final TMA [42]. The potential reasons for lower factor H in renal TMA with lupus nephritis, including autoantibodies against factor H or factor H gene mutations, need further study.

Furthermore, we divided TMA patients into two groups based on C4d deposition in the kidney and the value of serum factor H, and found that the patients with both C4d deposition and decreased serum complement factor H presented with higher pathological AI scores and poorer renal outcome. The results supported that over-activation of both complement classic and alternative pathways might aggravate TMA injury in lupus nephritis. Previous studies also suggested that classic pathway activation can recruit the potent components to further amplify generation of C3 and C5 activation products and alternative pathway activation might hold the key to continuous tissue damage via the amplification loop in kidney *situ *in lupus nephritis [43].

Tissue factor is an inducer of thrombosis. Interestingly, recent studies strengthened the idea that tissue factor activation which can be induced by the activation of complement, might be important in the pathogenesis of TMA [44,45].

We also observed that the ratio of nephrotic syndrome and the amount of proteinuria were both higher in the TMA group than that in the pure lupus nephritis group. As proteinuria might be a risk factor for thromboembolism, owing to loss of plasma antithrombin III and activation of the coagulation system [46,47], further studies were needed to confirm the pathogenetic role of proteinuria in renal TMA.

Many studies, including experimental models [48,49] and clinical observations [50], have shown complement activation to be essential in TMA. So our findings hold promise for complement inhibition as a therapeutic approach in the further treatment of TMA with lupus nephritis.

## Conclusions

In conclusion, there were various causes of renal TMA in lupus nephritis. TMA was an independent risk factor for renal outcome in lupus nephritis. Our study highlights the status of both complement classic and alternative pathway activation in the pathogenesis of renal TMA in lupus nephritis.

## Abbreviations

ADAMTS-13: a disintegrin and metalloprotease with thrombospondin type I repeats 13; aHUS: atypical hemolytic uremic syndrome; AI: activity indices; ANA: antinuclear antibodies; APS: anti-phospholipid syndrome; CFH: complement factor H; CI: chronicity indices; ds-DNA: double-stranded DNA; ELISA: enzyme-linked immunosorbent assay; ENA: extractable nuclear antigen; ESRD: end stage renal disease; H&E: hematoxylin and eosin; HR: hazard ratio; MBL: mannose-binding lectin; Meth: silver methenamine; PAS: periodic acid-Schiff; SLE: systemic lupus erythematosus; SLEDAI: systemic lupus erythematosus disease activity index; SNPs: single nucleotide polymorphisms; TMA: thrombotic microangiopathy; TTP-HUS: thrombotic thrombocytopenic purpura-hemolytic uremic syndrome.

## Competing interests

The authors declare that they have no competing interests.

## Authors' contributions

DS performed sample tests, analyzed data and wrote the manuscript. LHW and GL performed renal histopathological evaluation. FMW, XWY and DZ performed sample tests. MC collected clinical data on patients. FY recruited patients, collected samples and collected clinical data from patients, and designed and directed the study. MHZ designed and directed the study. All authors read and approved the manuscript for publication.

## Supplementary Material

Additional file 1 **Figure S1. Renal TMA identified by electron microscopy**. (**A**) Electron micrograph showed glomerular endothelial cell proliferation with narrowed capillary lumen, and widening of subendothelial space with electron dense deposits and infiltration of monocyte (EM × 5,000). (**B**) Higher magnification of part of Figure A, subendothelial widening with lucent area and electron dense deposits (EM × 20,000). Click here for file

Additional file 2 **Figure S2. Renal TMA identified by light microscopy and C4d staining on renal vessels**. (**A-C**)Thrombotic microangiopathy superimposed on lupus nephritis: (A) Glomerular endocapillary hypercellularity with intraluminal thrombus (Masson's trichrome ×400). (B) Thrombosis in interlobular arteriole (Periodic Acid-Silver Methenamine and Masson's trichrome ×400) (C) The thickened arteriole with swelling of endothelial cells and intimal fibrosis (Periodic Acid-Silver Methenamine and Masson's trichrome ×400). (**D**) C4d is positive beneath the vascular endothelium and within the basement membrane around the medial myocytes in patient with lupus nephritis (Original magnification ×400). Click here for file

Additional file 3 **Table S.1 Multivariate survival analysis of patients' renal prognosis with lupus nephritis**. Renal TMA and serum creatinine value were independent prognostic factors for renal survival. Click here for file
