# Supplementary figures and images for: The spectrum of renal thrombotic microangiopathy in lupus nephritis
Source: Arthritis Res Ther. 2013 Jan 15;15(1):R12. doi: 10.1186/ar4142 (PMC3672792; doi:10.1186/ar4142)

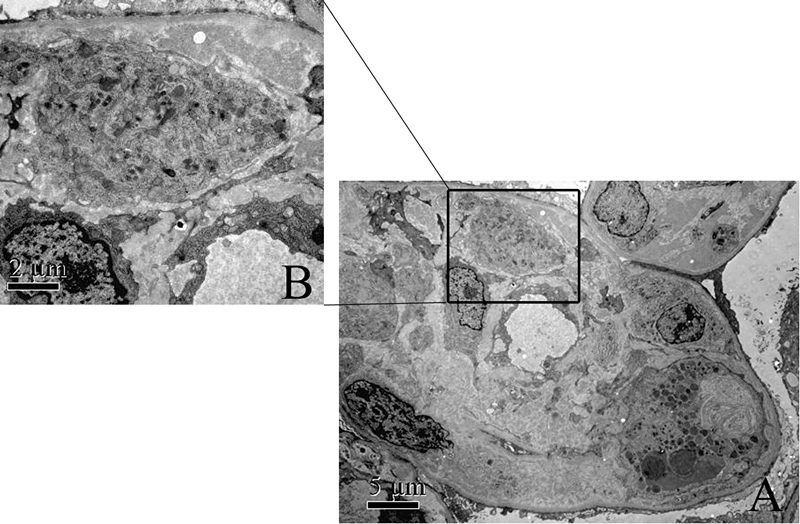

Supplement: Additional file 1 — Figure S1. Renal TMA identified by electron microscopy. (A) Electron micrograph showed glomerular endothelial cell proliferation with narrowed capillary lumen, and widening of subendothelial space with electron dense deposits and infiltration of monocyte (EM × 5,000). (B) Higher magnification of part of Figure A, subendothelial widening with lucent area and electron dense deposits (EM × 20,000). [file ar4142-S1.JPEG]

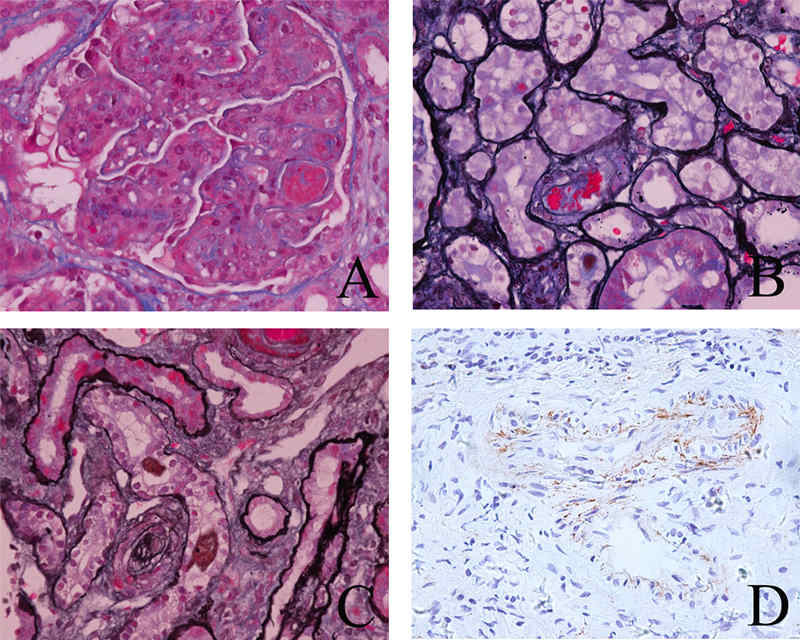

Supplement: Additional file 2 — Figure S2. Renal TMA identified by light microscopy and C4d staining on renal vessels. (A-C)Thrombotic microangiopathy superimposed on lupus nephritis: (A) Glomerular endocapillary hypercellularity with intraluminal thrombus (Masson's trichrome ×400). (B) Thrombosis in interlobular arteriole (Periodic Acid-Silver Methenamine and Masson's trichrome ×400) (C) The thickened arteriole with swelling of endothelial cells and intimal fibrosis (Periodic Acid-Silver Methenamine and Masson's trichrome ×400). (D) C4d is positive beneath the vascular endothelium and within the basement membrane around the medial myocytes in patient with lupus nephritis (Original magnification ×400). [file ar4142-S2.JPEG]
