# Supplementary material for: The spectrum of renal thrombotic microangiopathy in lupus nephritis
Source: Arthritis Res Ther. 2013 Jan 15;15(1):R12. doi: 10.1186/ar4142 (PMC3672792; doi:10.1186/ar4142)
Supplement: Additional file 3 — Table S.1 Multivariate survival analysis of patients' renal prognosis with lupus nephritis. Renal TMA and serum creatinine value were independent prognostic factors for renal survival. [file ar4142-S3.DOC]

**Additional file 3**

**Table S1 Multivariate survival analysis of patients’ renal prognosis with lupus nephritis**

|  |  | *HR* | *95% confidence interval* | | *P-value* |
| --- | --- | --- | --- | --- | --- |
| Step 1 | Age | 0.967 | 0.911 | 1.027 | 0.273 |
|  | Sex | 0.262 | 0.079 | 0.869 | 0.029 |
|  | Serum creatinine value | 1.002 | 1.000 | 1.004 | 0.074 |
|  | Proteinuria | 0.987 | 0.890 | 1.094 | 0.799 |
|  | Activity indices (AI)  Score | 1.115 | 0.960 | 1.294 | 0.154 |
|  | Renal TMA | 2.635 | 0.870 | 7.976 | 0.086 |
| Step 2 | Age | 0.966 | 0.910 | 1.027 | 0.268 |
|  | Sex | 0.273 | 0.086 | 0.870 | 0.028 |
|  | Serum creatinine value | 1.002 | 1.000 | 1.004 | 0.072 |
|  | Activity indices (AI)  Score | 1.110 | 0.961 | 1.281 | 0.156 |
|  | Renal TMA | 2.506 | 0.884 | 7.107 | 0.084 |
| Step 3 | Sex | 0.294 | 0.091 | 0.949 | 0.041 |
|  | Serum creatinine value | 1.002 | 1.000 | 1.004 | 0.015 |
|  | Activity indices (AI)  Score | 1.107 | .965 | 1.269 | 0.146 |
|  | Renal TMA | 2.317 | .830 | 6.471 | 0.109 |
| Step 4 | Sex | 0.358 | 0.116 | 1.106 | 0.074 |
|  | Serum creatinine value | 1.003 | 1.002 | 1.005 | <0.001 |
|  | Renal TMA | 2.772 | 1.009 | 7.617 | 0.048 |
